# Pericytes in Notch3 knockout and diabetic mice form engorged connections with vascular endothelial cells

**DOI:** 10.61340/pn3k1dm

**Published:** 2025-05-08

**Authors:** Timothy E. Vanderleest, Harper B. Gordon, Michael O’Hare, Philip Seifert, Joseph F. Arboleda-Velasquez

**Affiliations:** 1. Schepens Eye Research Institute of Mass Eye and Ear and the Department of Ophthalmology at Harvard Medical School, Boston, Massachusetts 02114, USA.

**Keywords:** Notch3, diabetic retinopathy, ultrastructure, image analysis, retinal capillaries, pericyte–endothelial cell interactions

## Abstract

**Purpose:**

Pericytes, cells crucially important to maintain a healthy microvasculature, make direct connections with vascular endothelial cells, yet the functional significance of these contacts remains largely unexplored. This study aims to investigate the ultrastructural morphological changes that occur in the interactions between pericytes and endothelial cells in mice lacking the Notch3 receptor and in diabetic retinopathy.

**Methods:**

Serial section transmission electron microscopy (ssTEM) was used to image mouse retinal ganglion cell layer capillaries in wild type (WT; 19 vessels), Notch3 knockout (KO; 16 vessels), conditional Notch3 KO (23 vessels), and diabetic mice (18 vessels). Over 2,000 images were manually segmented to trace the boundaries of the basement membrane, endothelial cells, mural cells, and peg-and-socket connections. Automated image analysis was used to measure contact lengths between pericytes and endothelial cells and peg-and-socket features.

**Results:**

While the vessels analyzed in each group were of similar diameter and pericyte coverage, Notch3 KO vessels had deeper pegs and increased connectivity between pericytes and endothelial cells. In both Notch3 KO and diabetic conditions, there was also an increase in the size of pericyte pegs.

**Conclusions:**

As the Notch3 receptor plays an important role in cell signaling between pericytes and endothelial cells, and diabetes is also known to disrupt Notch3 signaling, our hypothesis for the enlarged peg phenotype is that the pericytes and endothelial cells actively increase their contact surface to compensate for loss of Notch3 signaling.

## Introduction

Diabetic retinopathy (DR) is estimated to affect 9.6 million people in the US and is the leading cause of blindness among working-age adults in the US.^1^ Early changes in DR include basement membrane thickening and pericyte loss, altering the endothelial cell/pericyte ratio from 1:1 to 4:1.^2^ A large body of evidence suggests that pericyte loss leads to vascular pathological features including microaneurysms, microhemorrhages, acellular capillaries, and capillary nonperfusion.^3–8^

Cerebral autosomal dominant arteriopathy with subcortical infarcts and leukoencephalopathy (CADASIL) is a neurological disorder characterized by cerebral small vessel disease (SVD), adult-onset stroke, and vascular cognitive impairment and dementia (VCID) caused by mutations in NOTCH3.^9^ The hallmark features of CADASIL are granular osmiophilic membrane deposits and mural cell loss.^10–18^

What CADASIL and DR have in common, besides pericyte loss, is that both diseases involve Notch3 signaling. Notch3 encodes a transmembrane receptor, expressed mainly in mural cells (pericytes and smooth muscle cells), that plays a critical role in cell signaling pathways that support the function and survival of these cell types. In the case of CADASIL, there are specific mutations in the NOTCH3 gene, whereas in DR, cell culture and animal model studies have shown a downregulation of Notch3 signaling.^19, 20^

It remains unknown where exactly cell signaling takes place between endothelial cells (ECs) and pericytes. However, electron microscopy studies of capillaries in various organs have revealed that pericytes and ECs make direct cell connections through openings in the basement membrane. These connections between pericytes and endothelial cells manifest in different forms such as adherent plaques, contacts resembling gap junctions, and peg-and-socket junctions where the pericyte forms finger-like protrusions into EC invaginations (and less frequently, the EC protrudes into pericyte invaginations).^21, 22^ These contact sites are likely points of cell communication, including Notch3 signaling. In this study, we sought to examine the ultrastructure of pericyte-EC connections to understand whether they are altered when Notch3 signaling is disrupted, which ultimately leads to pericyte loss and vascular instability. We predicted that we would find a reduction in pericyte-EC direct connections and peg-and-socket junctions.

## Materials and methods

### Transmission electron microscopy methods

Mouse eyes were enucleated and brain tissue immersion (or pressure-perfusion) fixed with half-strength Karnovsky’s fixative (2 % formaldehyde + 2.5 % glutaraldehyde, in 0.1 M sodium cacodylate buffer, pH 7.4; Electron Microscopy Sciences, Hatfield, Pennsylvania) at room temperature.^23^ An eyecup was created from each eye by dissecting away the anterior from the posterior ends. The posterior eyecup and brain samples were then placed back into the half strength Karnovsky’s fixative for a minimum of 24 hours under refrigeration. After fixation, samples were rinsed with 0.1 M sodium cacodylate buffer, post-fixed with 2 % osmium tetroxide in 0.1 M sodium cacodylate buffer for 1.5 hours, *en bloc* stained with 2 % aqueous uranyl acetate for 30 minutes, then dehydrated with graded ethyl alcohol solutions, transitioned with propylene oxide, and infiltrated in tEPON-812 epoxy resin (Tousimis, Rockville, Maryland) utilizing an automated EMS Lynx 2 EM tissue processor (Electron Microscopy Sciences, Hatfield, Pennsylvania). The processed samples were oriented into tEPON-812 epoxy resin inside flat molds and polymerized using a 60 °C oven. Semi-thin sections were cut at 1 μm thickness through the mid-equatorial plane, intersecting the optic nerve in the posterior eyecup samples and stained with 1 % toluidine blue in 1 % sodium tetraborate aqueous solution for assessment by light microscopy.

A region containing a density of retinal ganglion cell layer (GCL) capillaries was selected from each retina sample from the semi-thin toluidine-blue-stained sections and block face trimmed to <1mm × 0.5 mm for ultramicrotomy. The block face edges were applied with a glue consisting of 50 % xylenes and 50 % rubber contact cement.^24^ Serial ultrathin-section (80 nm) ribbons were cut from each block face using a Leica EM UC7 ultramicrotome (Leica Microsystems, Buffalo Grove, IL, USA) and a diamond knife, using a hair tool to separate a set of sections within the ribbon and a fine forceps connected to a micromanipulator (DelSci Machine, Clayton DE) to collect onto 2×1 mm, single-slot, formvar-carbon-coated grids (Electron Microscopy Sciences, Hatfield, Pennsylvania).

After collection, each grid was air-dried and documented for serial section number and grid identification. A total minimum thickness of 2,000 nm was serial sectioned and collected within each block face sample. The ultrathin sections on grids were stained with aqueous 2.5 % gadolinium triacetate and modified Sato’s lead citrate^25^ using a modified Hiraoka grid staining system.^26^ Serial sections on grids were imaged using a FEI Tecnai G2 Spirit transmission electron microscope (FEI, Hillsboro, Oregon) at 80 kV interfaced with an AMT XR41 digital CCD camera (Advanced Microscopy Techniques, Woburn, Massachusetts) for digital TIFF file image acquisition. TEM imaging of vessels in the GCL layers of the retina was performed, and digital images were captured at 9300× magnification, 2k×2k pixel @16-bit resolution. Every GCL capillary selected was imaged throughout the serial section series and grids. Sections not collected or capillaries not observed and imaged due to artifacts of thin section folding or obscured by the edge of the single slot grid were documented.

### Animal model

Animal procedures were approved by the Institutional Animal Care and Use Committee of Massachusetts Eye and Ear and were performed in accordance with the Association for Research in Vision and Ophthalmology Statement for the Use of Animals in Ophthalmic and Vision Research.

### Diabetes model generation

Diabetes was induced in male C57BL/6J mice through multiple intraperitoneal injections of streptozotocin (50 mg/kg) administered over five consecutive days, following the protocol by Feit-Leichman et al.^27^ Mice with blood glucose levels exceeding 250 mg/dL were classified as diabetic. NPH insulin (0.1–0.3 units) was administered subcutaneously twice a week, as needed, when blood glucose levels surpassed 250 mg/dL, to prevent weight loss.

### Generation of Notch3 conditional knockout and eGFP

We generated a conditional knockout mouse to determine tissue-specific requirements for Notch3 expression beyond embryonic changes. The knock-in NOTCH3 vector contained LoxP–hNotch3 cDNA–IRES–EGFP–PolyA–LoxP–mCherry–PolyA (Supplemental Fig. 1). Human NOTCH3 sequence was used for plasmid construction; hence, the resulting mouse is humanized.

The linearized vector was subsequently delivered to ES cells (C57BL/6) via electroporation, followed by drug selection, PCR screening, and Southern blot hybridization. Chimeras generated through embryo injections of ES cell clones were crossed to C57BL/6 mice to obtain germline transmission. The resulting NOTCH3 inducible knockout mice (WT76 line) were viable and fertile and displayed no gross abnormalities. These mice, crossed to Cre-ERT2, have strong tamoxifen-inducible Cre activity in all tissue types. Induction of Cre recombination is initiated with 4-hydroxytamoxifen intraperitoneal injection of 500 μg of tamoxifen in 200 μL of corn oil daily for three days. Recombination between LoxP sites is catalyzed by Cre recombinase leading to the removal of the LoxP-flanked NOTCH3 and the expression of mCherry.

### Immunohistochemistry for Notch3 conditional KO

Processing and immunolabeling of mouse retinal whole mounts were performed as previously described.^28^ Briefly, eyes were enucleated and fixed in 4 % paraformaldehyde for 2 hours at 4 °C. Retinas were incubated with 1 % bovine serum albumin, 0.1 % Triton X-100, and 3 % donkey serum overnight at 4 °C, incubated with isolectin B4 (IB4) (1:200; Thermo Fisher Scientific), and α-smooth muscle actin (α-SMA) (1:200; Thermo Fisher Scientific), followed by Alexa Fluor 568 (1:200; Thermo Fisher Scientific). While the Alexa 568 does have spectral overlap with mCherry expressed by the lox/Cre model, mCherry signal is weak in this fixed tissue and overpowered by the Alexa 568. Confocal images were acquired using a Leica SP8 (Leica Microsystems).

### Image segmentation and analysis of TEM data

TEM images were manually segmented by tracing boundaries of the cells, lumen, and basement membrane via ImageJ (or FIJI) v4 drawing tools. The boundaries were then filled in to create a six-class segmentation of pericytes, pericyte pegs, endothelial cells, basement membrane, lumen, and background. The segmentation was used for all the measurements described below.

Measurements of morphological properties from the segmented images were performed using MATLAB R2021a. Vessel diameter, *D*, was calculated using the measurement of the vessel circumference, *C*, and the formula for a circle: *D* = *C* / π. The circumference of the vessel was measured as the length of the outer perimeter of the basement membrane using the *regionprops* function in the MATLAB Image Processing Toolbox. Pericyte volume % and basement membrane volume % are percentages of the total volume of the vessel excluding the lumen.

To measure pericyte coverage length and pericyte–endothelial cell contact length, we developed an automated algorithm, using tools from the MATLAB Image Processing Toolbox, that operates on the segmented images. The first step of the algorithm was to generate a skeletonized representation (using MATLAB function *bwskel*) of the basement membrane and the pericyte–endothelial cell contacts. A mask of the pericyte–endothelial cell contacts was generated by using a morphological dilation of the endothelial cell mask by 1 pixel and finding the pixels which overlap with the pericyte mask. The next step was to classify each pixel of the skeletonized image into one of three classes: lines of cell–cell contact (Class 1), lines of pericyte coverage but no cell contact due to the presence of basement membrane (Class 2), and lines of no coverage (Class 3). If the pixel was on the outer perimeter of the skeleton, it was assigned to Class 3; Class 1 pixels were already determined by the mask as described above, and all remaining pixels were assigned to Class 2. Lengths for each class were measured by summing the number of skeletonized pixels in the class.

Pericyte peg maximum depth was measured from the segmented images using the following automated algorithm. A mask of the endothelial cell, pericyte pegs (i.e., just the part protruding into the endothelial cell), and the lumen was generated from the segmented image. A morphological closing (MATLAB function *imclose*, using a 134 nm radius disk-shaped structuring element) was performed on the mask to fill in possible small gaps between the pericyte peg and the endothelial cell. A distance transform (MATLAB function *bwdist*) was then performed using the region outside the mask (i.e., the complement of the mask), thus giving a map of distances from the abluminal surface of the endothelial cell that increase moving toward the lumen. For each pericyte peg, distance map values were extracted from the pegs’ mask region and the maximum distance value was found.

### Quantification of α-SMA coverage from immunofluorescence image data

In the α-SMA images, the vasculature was detectable at low intensities. To get a mask of the vessels, we first applied a Top-hat filter for localized background subtraction and then applied a fixed low-intensity threshold. To get a mask of the α-SMA coverage we applied automated thresholding (Otsu’s method) to the Top-hat filtered images. Using a custom MATLAB algorithm, we subdivided the vessel mask into branches and measured the average diameter of each branch. For each branch, α-SMA coverage % was also measured, which was defined as the area of the α-SMA mask on the branch divided by the branch area multiplied by 100.

### Statistical analysis

Statistics were performed using GraphPad Prism (Version 10.2.2). Each dataset was tested for normality using the D’Agostino & Pearson, Anderson Darling, Shapiro-Wilk, and Kolmogorov-Smirnov tests. Unless otherwise noted in the legend, the Kruskal-Wallis test was used to test for significance. P < 0.05 was considered statistically significant.

## Results

**Figure 1.**
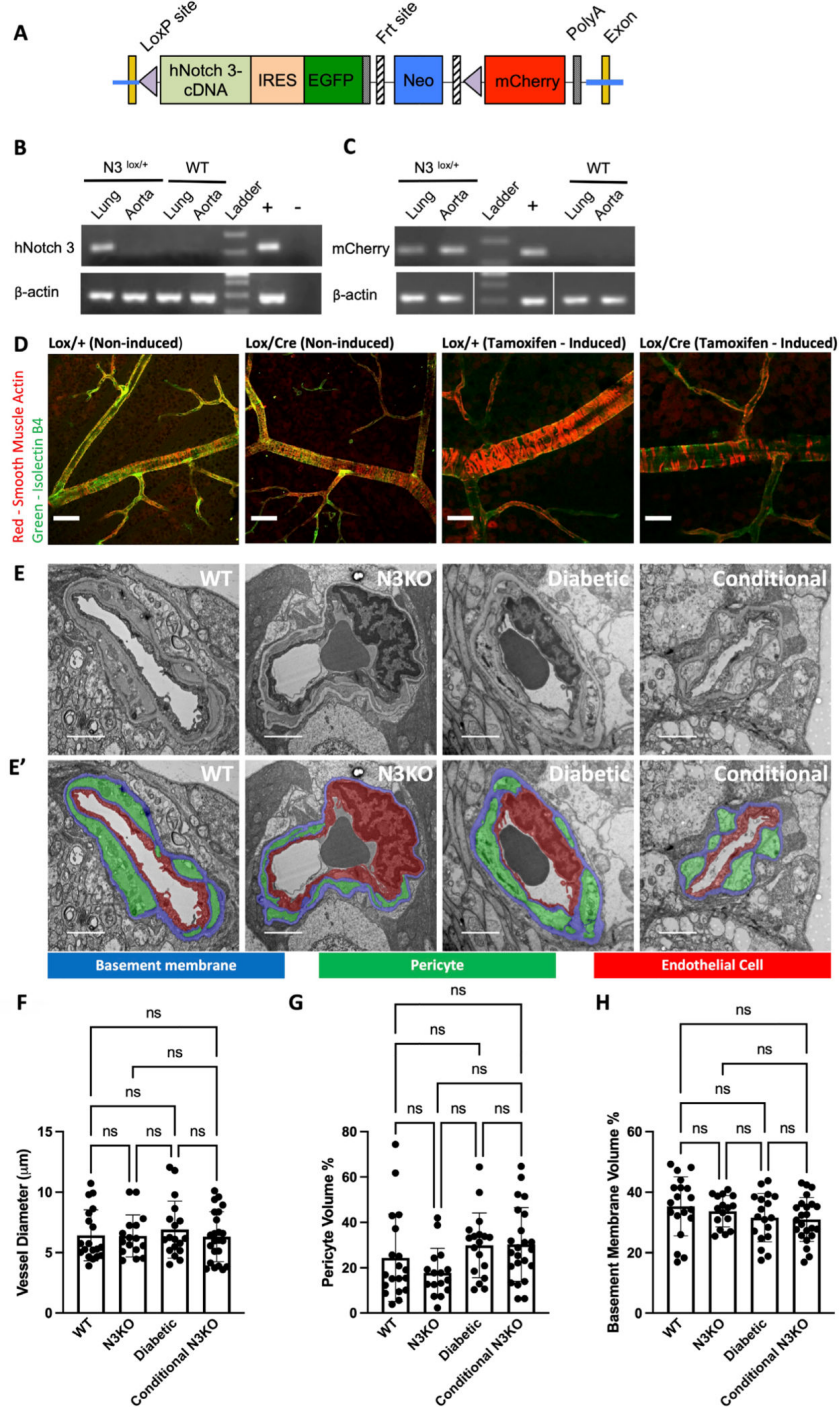
Generation of a conditional Notch3 KO and basic vessel properties. **(A)** Schematic of the targeted Notch3 locus. **(B)** Expression of the hNotch3 knock-in in lung from N3 ^lox/+^ mice by RT-PCR. Expression in the aorta was not detected consistent with preferential expression of Notch3 in small vessels. The positive control (+) was a mouse genome and the negative control (−) was no DNA. **(C)** Expression of mCherry after Cre-mediated removal of Notch3 transgene was confirmed by crossing to a β-actin Cre deleter mouse. Bottom samples were run in same gel but rearranged to match the top gel loading order (white lines). **(D)** At 12 months of age, mice, including homozygotes carrying the human Notch3 loxed allele (hN3 lox/hN3 lox) and heterozygotes with one loxed allele and one endogenous mouse Notch3 allele, demonstrate normal mural cell coverage in the retina. However, hN3 loxed mice harboring the Notch3 Cre Ert2 driver exhibit mural cell loss six months after tamoxifen-induced Notch3 deletion. Staining for alpha smooth muscle actin (red channel) and Isolectin B4 (green channel) highlights these observed changes in mural cell presence. **(E)** TEM images of WT, Notch3 KO, diabetic, and Conditional KO (from left to right) retinal blood vessels. **(E’)** TEM images with segmentation color overlay of endothelial cells (red), pericytes (green), and basement membrane (blue) for the images in (E). **(F)** Quantification of vessel diameters for each genotype. **(G)** Quantification of pericyte volume as a percent of the total vessel volume. **(H)** Quantification of basement membrane volume as a percent of total vessel volume. Scale bars are 25 microns in (D) and 2 microns in (E-E’). Mean ± S.D. in (F-H).

In addition to a developmental Notch3 knockout used for our analyses, we generated a novel Notch3 conditional knockout to study how pericyte–endothelial cell interactions change in mature capillaries when Notch3 signaling is disrupted (Fig. 1A–C). We report that conditional knockout of Notch3 in hN3 Cre/lox mice after tamoxifen induction at 6 months of age leads to a reduction in mural cell coverage by 12 months of age, as assessed by immunohistochemistry of α-SMA coverage (Fig. 1D).

Conversely, mice including non-injected hN3 Cr/lox, homozygotes carrying the human Notch3 loxed allele (hN3 lox/hN3 lox), and heterozygotes with one loxed allele and one endogenous mouse Notch3 allele, demonstrated normal VSMC cell coverage in the retinal arterioles at 12 months of age (Fig. 1D). Additionally, we found that the reduction in α-SMA coverage was vessel diameter dependent, with significant reductions in vessels larger than capillaries (Supplementary Fig. 2).

Given that there is mural cell loss in the vasculature of DR and Notch3-associated small vessel disease, we wanted to inspect the ultrastructure of these cells and their interactions with endothelial cells in capillaries where pericytes were still present. To do so, we acquired serial-section transmission electron microscopy (ssTEM) image data of retina capillaries in 6-month-old mice in wild type (WT), Notch3 KO, diabetes, and conditional Notch3 KO conditions (Fig. 1E). Image segmentation of the endothelial cell, pericyte, pericyte pegs, lumen, and basement membrane (Fig. 1E’) was the basis for all the following measurements and quantification.

The dataset of TEM images that we performed image segmentation on consisted of 19 WT, 18 diabetic, 16 Notch3 KO, and 23 conditional Notch3 KO vessels. For each vessel, there were a series of z-sections acquired, with ~28 images per vessel in WT (526 total images), ~33 images per vessel in Notch3 KO (528 total images), ~29 images per vessel in diabetic (516 total images), and ~20 images per vessel in the conditional KO (470 total images). The image data for each vessel consisted of a z-stack with z-resolution of ~80nm; however, there were some images which were excluded due to poor image quality or imaging artifacts.

We first checked basic properties of the vessels such as diameter and the amount of pericyte cells present on the vessel. In each condition, the vessel diameters were consistent with capillaries (~4–12 μm) and not significantly different in size from each other (Fig. 1F). Next, to determine how much pericyte was present on each vessel, we measured the percentage of pericyte voxels to total vessel voxels (excluding voxels classified as lumen, which in some cases contained erythrocytes). We found that the proportion of pericyte of the capillary volume was not significant between our different groups (Fig. 1G). Lastly, we were interested in the proportion of basement membrane in the vessels due to the well-established finding of basement membrane thickening in CADASIL and DR^29–31^, however there was no difference between the groups (Fig. 1H).

### Pericyte–endothelial cell contact length

**Figure 2.**
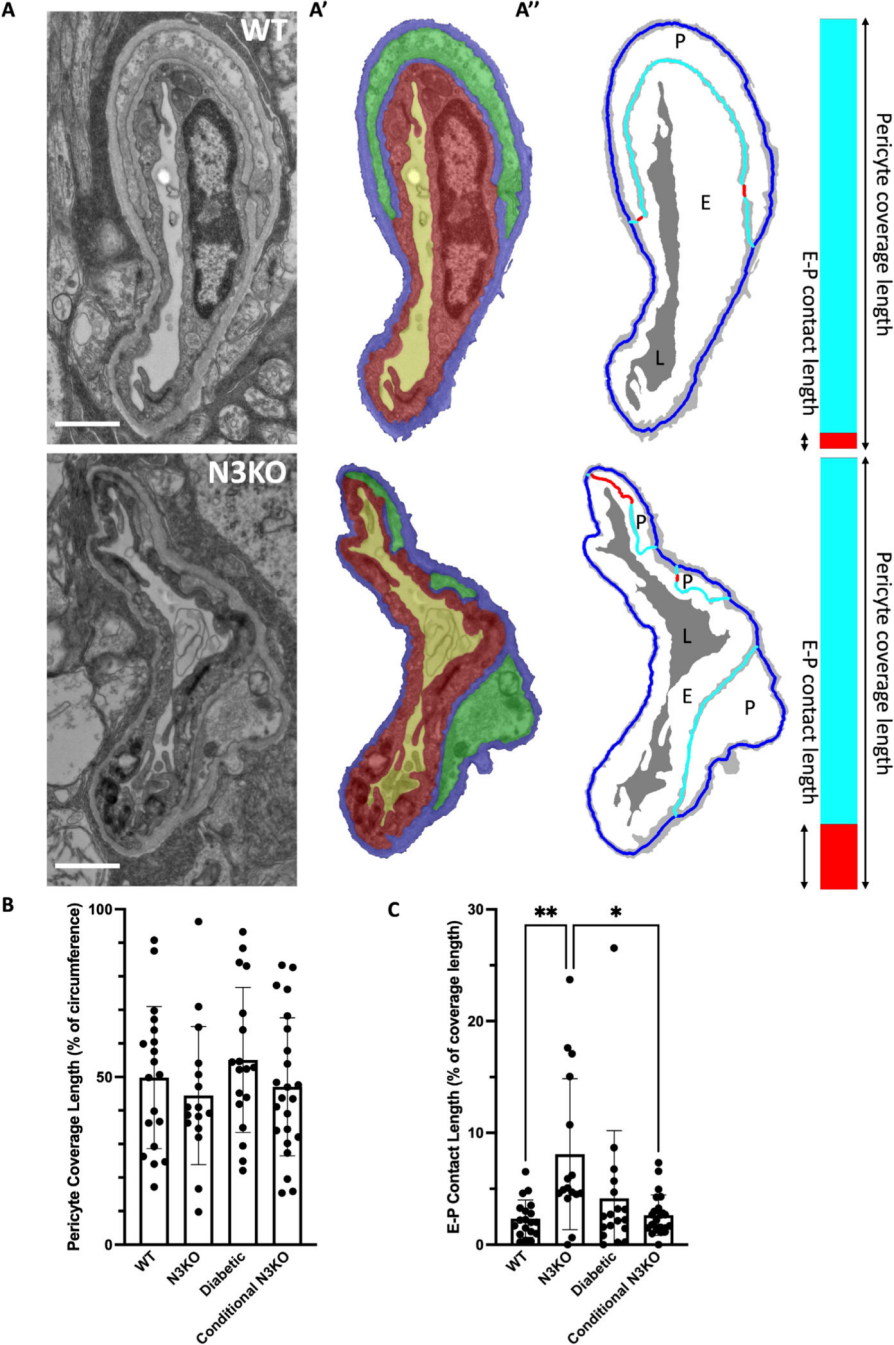
Notch3 KO pericytes form more connections with endothelial cells. **(A)** Raw TEM images (WT on top and Notch3 KO on bottom) of a capillary. **(A’)** Color overlay of the segmentation of the basement membrane (blue), pericyte (green), endothelial cell (red), and lumen (yellow) on the images in (A). **(A’’)** Skeletonization of the basement membrane (cyan for membrane between the cells and dark blue elsewhere) and direct endothelial–pericyte (E-P) cell contacts (red) overlayed on an image showing the basement membrane in gray and the lumen in dark gray. Skeletonized lines were dilated for visibility. Stacked bar graph on the right shows the ratio of direct cell contact (red) to total coverage length (red and cyan). **(B)** Quantification of pericyte coverage as a percent of the vessel circumference. **(C)** Quantification of direct contact length as a percentage of pericyte coverage length. Scale bars in (A) are 1 micron. Mean ± S.D. in (B–C). ** denotes P<0.01 and * denotes P<0.05.

After we found that the vessels in each group had similar proportions of pericyte to total vessel volume (Fig. 1G), we next wanted to know how frequently pericytes and endothelial cells make direct contact with each other (Figs. 2A–A”; places where green and red colored pixels meet). To account for the bias that vessels with more pericyte coverage have more potential places to make direct contact (Fig. 2A”; red line), we normalized contact length by pericyte coverage length (cyan and red line length sum; Fig. 2A”). First, we measured the amount of pericyte coverage for each vessel. While there was a notable amount of variability, each group had approximately 50 % coverage and did not differ significantly from each other (Fig. 2B). Surprisingly, however, when normalized by pericyte coverage and represented as a percentage, pericyte–endothelial cell contact length was significantly higher in Notch3 KO than WT (Fig. 2C, P = 0.0051). In diabetic vessels, the contact percentage was also increased compared to WT; however, the difference was not statistically significant. The conditional KO did not show a significant increase compared to WT.

Given that we did not see reduced pericyte coverage (Fig. 2B) or reduced volume % (Fig. 1G) in pathological conditions known for pericyte loss, we took another look at the TEM data to specifically inspect some of the larger diameter vessels (Supplemental Fig. 3). We performed image segmentation on 3 WT vessels and 4 conditional Notch3 KO vessels. Combined with the capillaries previously segmented, we found a significant positive correlation in WT (*R*^2^ = 0.66) and an unsignificant correlation in the Conditional N3KO (*R*^2^ = 0.01; Supplemental Fig. 3C). When restricting the data to vessels larger than 10 microns in diameter (N=4 in WT and N=5 Conditional N3KO), there was significantly reduced coverage in the conditional N3KO (Supplementary Fig. 3D).

### Peg-and-socket properties

**Figure 3:**
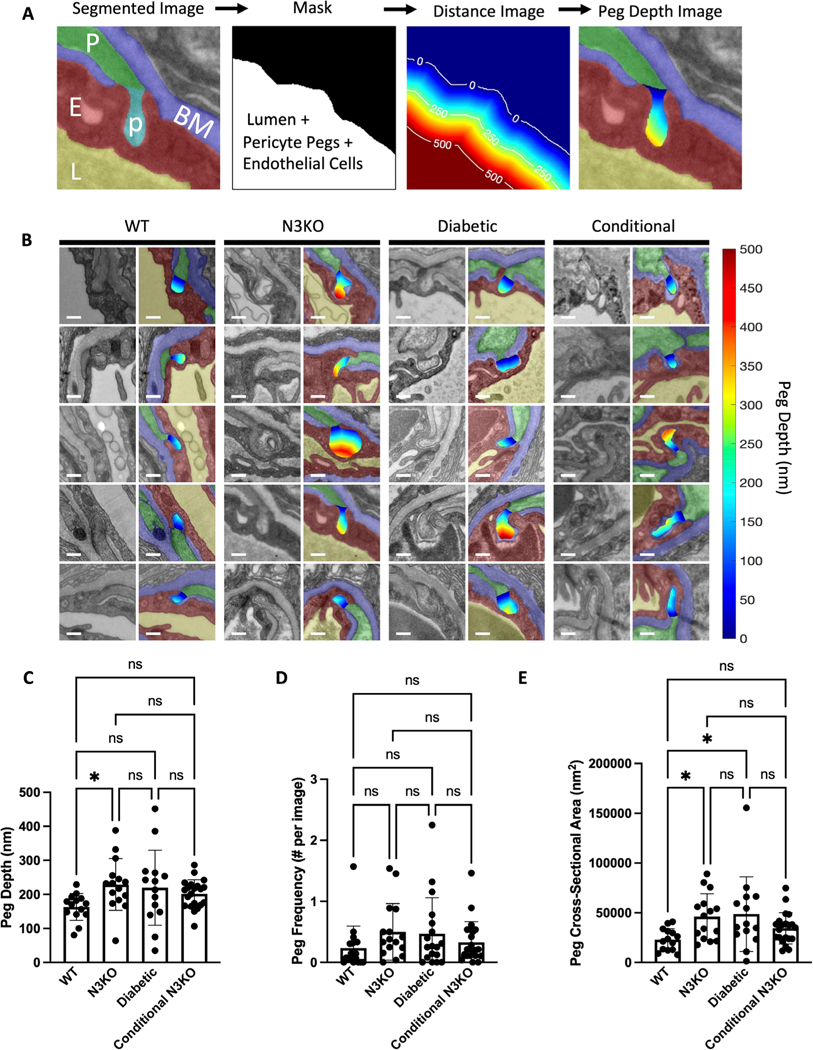
Notch3 KO pericytes form deeper peg & socket connections with endothelial cells. **(A)** Illustration of our peg depth measurement method. A Color overlay image (left panel) shows the segmented regions: endothelial cell (E; red), pericyte (P; green), pericyte pegs (p; cyan), basement membrane (BM; blue), and lumen (L; yellow). The segmentation is used to generate a mask of the endothelial cell, lumen, and pericyte pegs (second panel). A distance transform is used to map distance from outside the mask inward (third panel, contour lines show distances in nm), and the mask of the pericyte pegs are used to extract distance values (opaque colored region in fourth panel). **(B)** Raw TEM images (left columns) and TEM with pericyte pegs colored according to their protrusion depth into the endothelial cell. Scale bars are 250 nm. **(C)** Quantification of pericyte peg maximum depth. **(D)** Quantification of pericyte peg frequency (in number of peg components per image). **(E)** Quantification of pericyte peg cross-sectional area. Mean ± S.D. in (C-E). * denotes P < 0.05.

Peg-and-socket junctions are another hallmark of the interaction between pericytes and endothelial cells, so we also wanted to investigate whether these features changed when Notch3 signaling was disrupted. One metric of interest was the maximum protrusion depth of pericyte pegs from the abluminal surface of the endothelial cell. Using a computational technique known as a distance transform, we were able to map distances from the abluminal surface of the endothelial cell onto the pericyte pegs (illustration of method in Fig. 3A; Fig. 3B shows examples from each condition). Interestingly, we observed more deep pegs in all the pathological conditions. However, the Notch3 KO group was the only one that showed statistical significance from WT (Fig. 3C; P = 0.0165). We did not find differences in the frequencies of pericyte pegs (Fig. 3D), but we found that Notch3 KO and diabetic pegs were significantly larger than WT (Fig. 3E; P = 0.0275 between WT and N3KO; P = 0.0470 between WT and Diabetic). In the conditional KO, the pegs were also larger on average; however, it was not different.

## Discussion

In this study, image segmentation and automated analysis were used to measure properties of the cellular interactions between pericytes and endothelial cells. Measurements from the vessel segmentation show that, in Notch3 KO, pericytes and endothelial cells form significantly more direct contact compared to WT, and pericyte peg-and-socket connections are larger and extend deeper into endothelial cells. One potential role of these peg-and-socket connections is that they act as a mechanical hook, allowing the pericyte to transmit forces to contract and regulate vascular tone or flow.^32^ Another potential role for peg-and-sockets is that they serve as a site for cell signaling, as it is usually a location where the cells make direct contact with each other. As the Notch3 receptor plays an important role in cell signaling between pericytes and endothelial cells, our hypothesis for the increased connectivity and enlarged peg phenotype is that the two cells actively increase their contact surface to compensate for the loss of Notch3 signaling.

In the capillaries that we analyzed via TEM in this study, we did not see an overt reduction in pericyte coverage in our DR model, Notch3 KO, or conditional Notch3 KO. This finding was unexpected, given previous reports of pericyte loss in these models. However, when examining retinal vessels larger than capillaries, we observed a significant reduction in mural cell coverage in the conditional Notch3 KO, shown both by immunofluorescence imaging via α-SMA coverage measurement (Supplementary Fig. 2) and through cell segmentation of a sample of larger vessels in TEM (Supplementary Fig. 3). These results could indicate that mural cell loss in capillaries is less overt than in larger arterioles and venules, or that it occurs at a later stage of disease progression. One caveat of the TEM data is that, although it is ideal for analyzing subcellular features, it is currently unsuitable for measuring large-scale properties such as pericyte numbers or densities along the vasculature.

In our DR model, the pericyte–endothelial cell contact length percentage was elevated compared to WT (an average of 4.0 % versus 2.3 % in WT), although this increase was not statistically significant. Interestingly, the DR pericyte peg-and-socket junctions were significantly enlarged compared to WT, with similar cross-sectional areas to the Notch3 KO on average. One might expect that larger pegand-socket junctions would entail higher direct cell–cell contact; however, there are other factors to consider such as the following: not all peg-and-sockets were points of direct cell–cell contact (as there occasionally is basement membrane between the cells), and there are many flat contacts that do not have peg-like protrusions (see Figs. 2A–A”). It could be that in DR there are fewer non-peg contacts or more basement membrane at peg-and-socket locations. Another interesting finding from our analysis was that we did not detect basement membrane thickening in our DR model, a common observation in DR studies. One possible explanation for this finding is that pericytes help to regulate the basement membrane, and because our DR vessels have similar amounts of pericyte coverage to WT, this could have prevented the effect of basement membrane thickening. For future studies, it might be interesting to investigate whether there is a relationship between localized basement membrane thickness and pericyte coverage.

In the conditional Notch3 KO, we observed an increase in pericyte–endothelial cell direct contact and larger pericyte pegs (~70 % larger than WT on average); however, neither of these differences was statistically significant. While we found no detectable phenotype using TEM, further investigation should examine inducing the KO at different time points to explore whether there is a differential effect during aging.

## Supplementary Material

1
