# Supplementary material for: Pericytes in Notch3 knockout and diabetic mice form engorged connections with vascular endothelial cells
Source: ScienceBank. Author manuscript; Available in PMC 2026 Feb 1. (PMC12860123; doi:10.61340/pn3k1dm)

Supplementary figures

Supplemental Figure 1. Schematic of vector targeting strategy for NOTCH3. **(1)** The wild type allele. **(2)** The targeting vector that includes LoxP–Notch3 cDNA–IRES–EGFP–PolyA–LoxP–mCherry–PolyA cassette. In the targeting vector, the Neo cassette will be flanked by Frt sites, and Notch3 cDNA will be flanked by LoxP sites. DTA will be used for negative selection. **(3)** The targeted allele. **(4)** The conditional KO allele will be obtained after Flp-mediated recombination. **(5)** The constitutive KO allele will be obtained after Cre-mediated recombination.

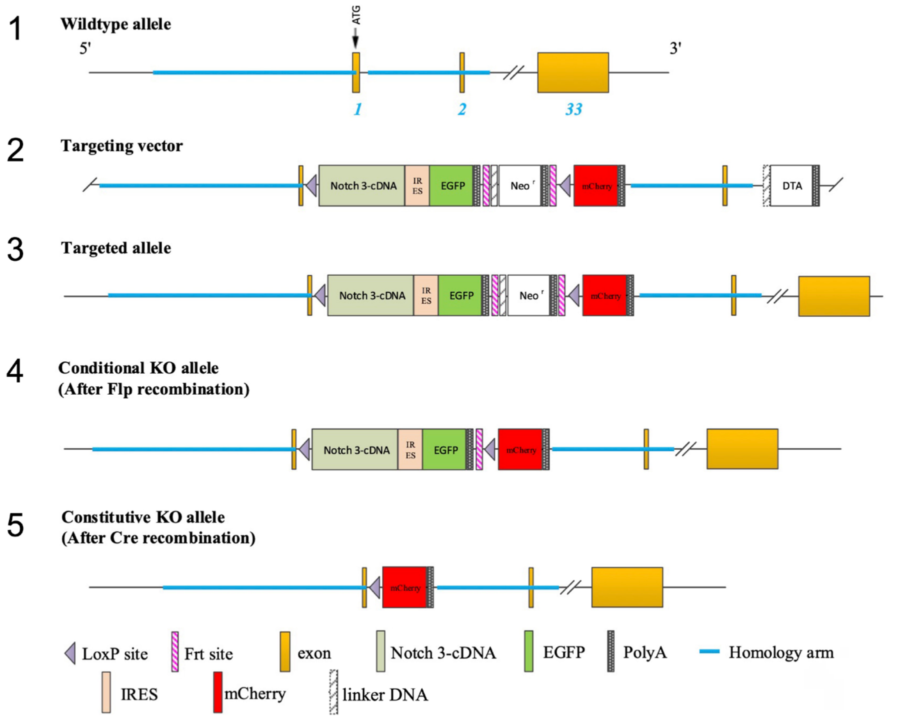

Supplemental Figure 2. Induction of Notch3 KO reduces α-SMA coverage in larger blood vessels.

(A–A') Immunofluorescence imaging of retinal vessels stained for α-SMA in control (A) and the induced Notch3 KO (A'). **(B–B')** α-SMA coverage was detected via an automated intensity threshold (Otsu's method; shown in cyan). The vessels were segmented using a low-intensity threshold (shown as the union of black and cyan regions). **(C-C')** Individual vessel branches were segmented and the average diameter of the branches were measured (shown here with branches colored according to the colorbar on the right). **(D)** Scatter plot of α-SMA coverage area % for each branch (defined as the cyan area divided by the vessel area in B–B') verses branch width in control (black circles) and the induced Notch3 KO (magenta squares). **(E–E''')** α-SMA coverage area % was compared for three difference branch diameter (d) bins: Small (d ≤ 5 pixels), Intermediate (5 < d ≤10), and Large (d > 10). * indicates p < 0.05. The Mann-Whitney test was used.

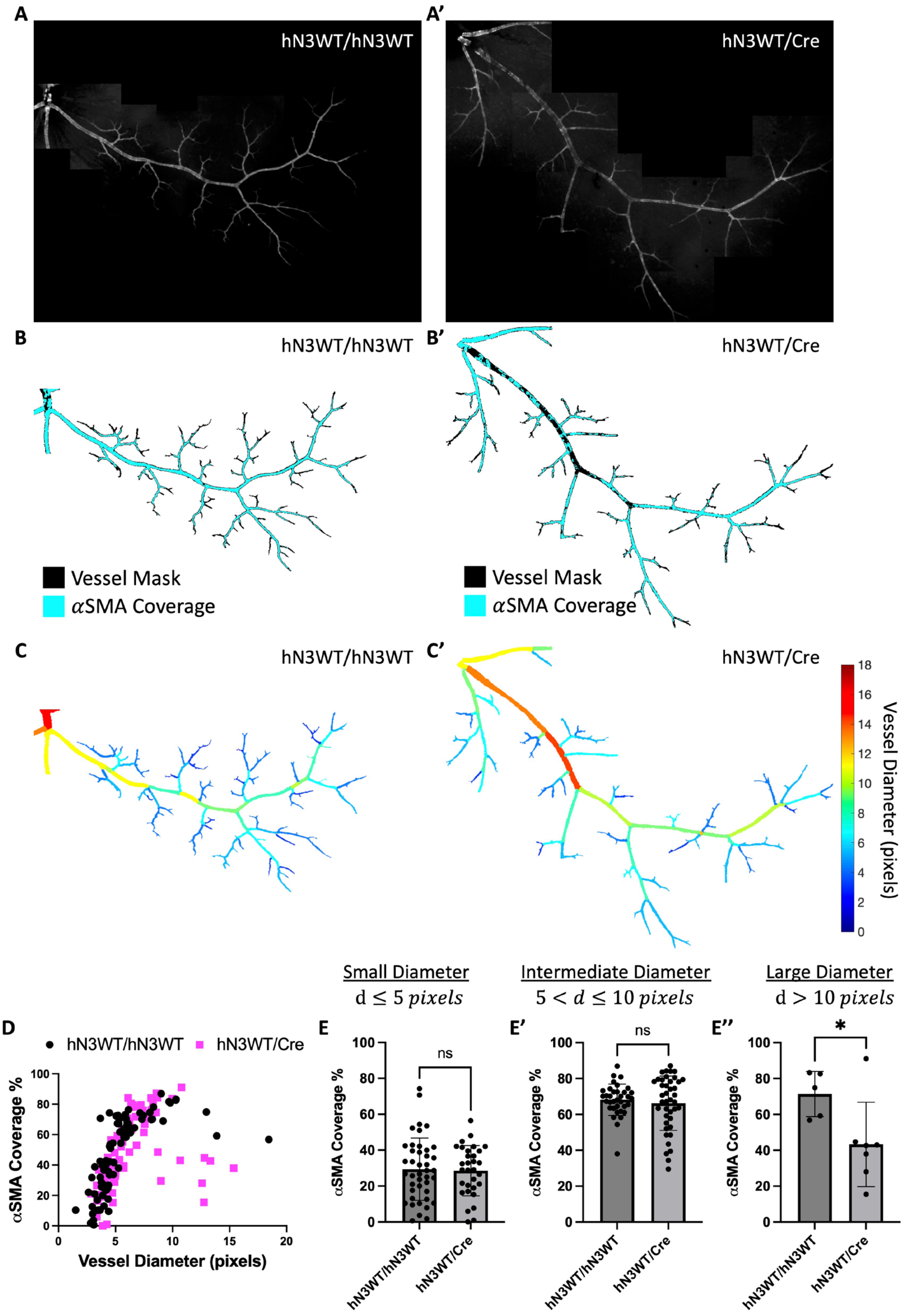

Supplemental Figure 3. Induction of Notch3 KO reduces the amount of mural cell coverage in larger blood vessels.

(A–A") Segmented TEM images for three examples of WT retinal blood vessels for vessels larger than capillaries. The color overlay shows basement membrane (blue), endothelial cells (red), and mural cells (green). **(B–B")** Segmented TEM images for three examples of conditional Notch3 KO retinal blood vessels for vessels larger than capillaries. **(C)** Scatter plot of pericyte coverage length % versus vessel diameter for WT (blue circles) and conditional Notch3 KO (magenta squares). All vessels (capillaries and larger) were included. Linear regression lines are plotted in the scatter plot (WT: R^2 = 0.66; conditional Notch3 KO: R^2 = 0.01). **(D)** Quantification of pericyte coverage length % for just the large vessels (larger than 10 microns). ** indicates P < 0.01 using the Mann Whitney test. The 10 μm scale bar applies to all images (A–A" and B–B").

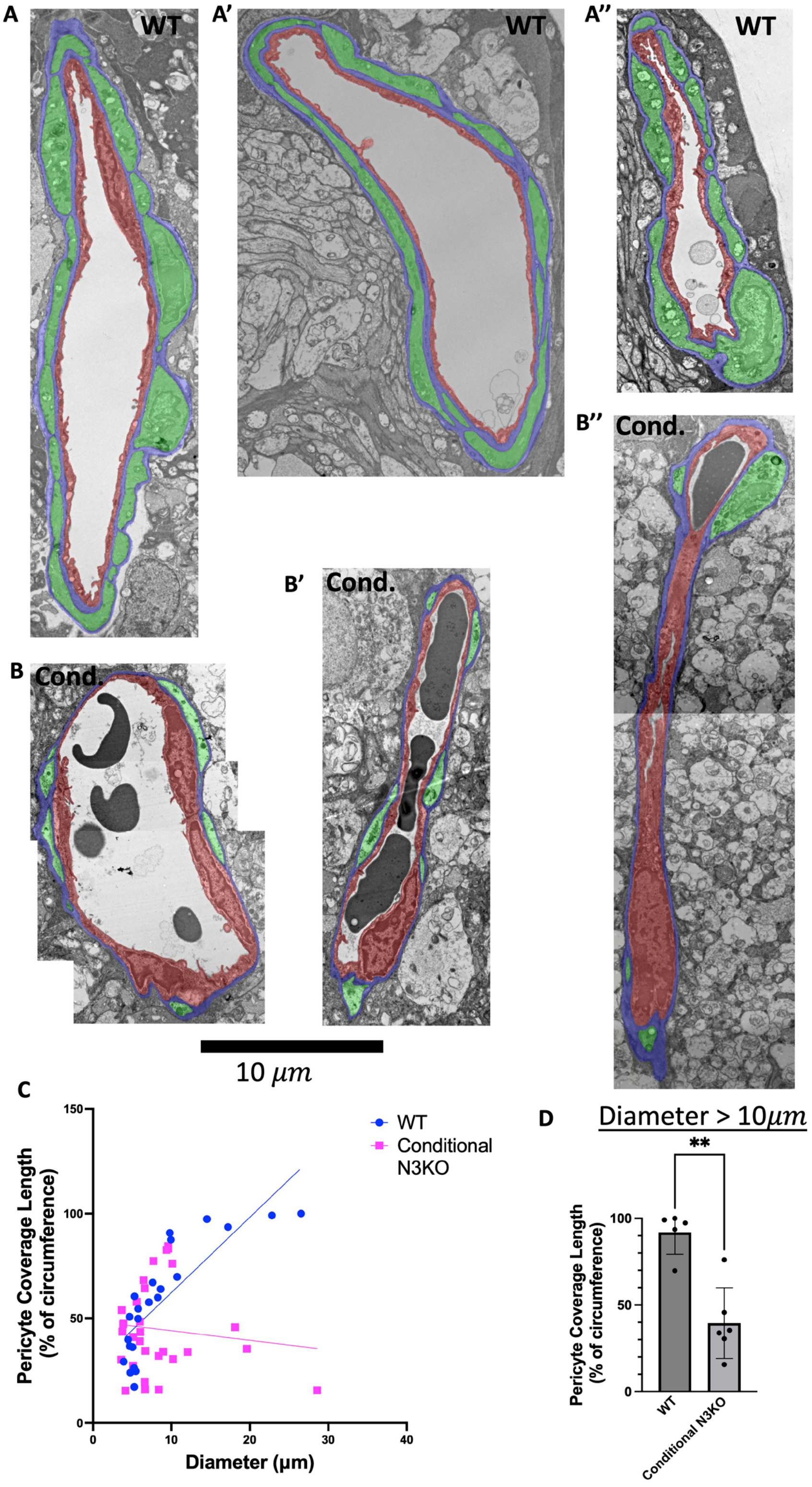

Supplement: 1 [file NIHMS2124968-supplement-1.pdf]
